# Quantum Clock Synchronization with a Single Qudit

**DOI:** 10.1038/srep07982

**Published:** 2015-01-23

**Authors:** Armin Tavakoli, Adán Cabello, Marek Żukowski, Mohamed Bourennane

**Affiliations:** 1 Physics Department, Stockholm University, S-10691, Stockholm, Sweden; 2 Departamento de Física Aplicada II, Universidad de Sevilla, E-41012 Sevilla, Spain; 3 Instytut Fizyki Teoretycznej i Astrofizyki, Uniwersytet Gdański, PL-80-952 Gdańsk, Poland

## Abstract

Clock synchronization for nonfaulty processes in multiprocess networks is indispensable for a variety of technologies. A reliable system must be able to resynchronize the nonfaulty processes upon some components failing causing the distribution of incorrect or conflicting information in the network. The task of synchronizing such networks is related to Byzantine agreement (BA), which can classically be solved using recursive algorithms if and only if less than one-third of the processes are faulty. Here we introduce a nonrecursive quantum algorithm, based on a quantum solution of the detectable BA, which achieves clock synchronization in the presence of arbitrary many faulty processes by using only a single quantum system.

In many multiprocess networks, including data transfer networks, telecommunications networks, the global positioning system, and long baseline interferometry, the individual processes need to have clocks that must be synchronized with one another^1^,^2^. To this purpose, individual processes' clocks must periodically be resynchronized. This motivates the need for clock synchronization algorithms which work despite the faulty behavior by some of the processes. Faulty behavior can occur due to a variety of causes, including crashing, transmission failure, and distribution of incorrect or inconsistent information in the network^3^. A clock synchronization algorithm should achieve the following tasks: C1) For any given instant, the time of all nonfaulty processes' clocks must be the same. This is necessary, but not sufficient, since simply stopping all clocks at zero satisfies C1. We therefore need to assume that a process' logical clock also keeps the rate of its corresponding physical clock. In addition, synchronizing may cause further errors, so we require that: C2) There is a small bound on the amount that a process' clock is changed during synchronization^4^.

Reliable clock synchronization algorithms can be complicated. To simplify the problem we shall work under the following assumptions^4^: A1) Initially, all clocks are synchronized to the same value. Physical clocks typically do not keep perfect time but drift with respect to one another. This motivates the following assumption: A2) All nonfaulty processes' clocks run at one second in clock time per second in real time. A general problem arises from the clocks continuously changing during the synchronization procedure. Unless the synchronization algorithm is very fast, this will cause problems. This motivates our last assumption: A3) A nonfaulty process can read the time difference between the clock of another process and its own.

A method to achieve synchronization is to use interactive consistency algorithms (ICAs) in which all nonfaulty processes reach a mutual agreement about all the clocks^4^. An ICA should satisfy that, for every process *p*: (1) Any two nonfaulty processes obtain the same value of process *p*'s clock, even if *p* is faulty. (2) If *p* is nonfaulty, then every nonfaulty process obtains the value of *p*'s clock. The synchronization problem can classically be solved using recursive algorithms if and only if less than one-third of the clock are faulty.

The conditions for an ICAs are similar to the ones of the problem of Byzantine Agreement (BA) in the case of which: (i) All nonfaulty processes obtain the same value and (ii) if process *p* is nonfaulty, then all nonfaulty processes obtain the value it sends^4^,^5^. Nevertheless, it has been shown that even quantum methods cannot solve the BA if one-third or more of processes are faulty^6^.

However, for most applications, including clock synchronization, it is sufficient to consider a scenario called detectable Byzantine agreement (DBA) or detectable broadcast^7^,^8^. In this case, conditions (i) and (ii) are replaced with: (i′) either all nonfaulty processes obtain the same value or all abort, and (ii′) if process *p* is nonfaulty, then either every nonfaulty process obtains the same value or aborts. By “abort” we mean treating the value as undefined and exiting the protocol.

Classical ICAs can only achieve DBA if less than one-third of the processes are faulty^4^ and agreement is achieved by majority voting using a recursive algorithm, called *OM*(*n*), where *n* is the number of faulty processes. The *OM*(*n*) algorithm works as follows. We label the processes as *P_k_*, with *k* = 1, 2, …, *m*. If *n* = 0, then *P*_1_ distributes its value to every other process. Every process uses the value received from *P*_1_ and, in case no value is obtained, uses 0. If *n* > 0, then *P*_1_ distributes its value to every other process. For *k* = 2, …, *m*, let *x_k_* denote the value obtained by *P_k_* from *P*_1_. If *P_k_* receives no message, then let *x_k_* = 0. *P_k_* acts as *P*_1_ in algorithm *OM*(*n* − 1) by distributing *x_k_* to the remaining *m* − 2 processes. For every *k* and for all *j* ≠ *k*, let *x_j_* be the value received by *P_k_* from *P_j_* using *OM*(*n* − 1), and in case no value was received *x_j_* = 0. *P_k_* decides on the value obtained from the median of (*x*_1_, …, *x_m_*). Thus, *OM*(*n*) requires *O*(*m^n^*^+1^) transmitted messages to solve the task.

The DBA is an example of a communication task for which quantum resources can provide a solution, while classical tools cannot. Nevertheless, the special case of DBA in a three process network with one faulty process, has been solved using quantum methods based on three-qutrit singlet states^7^,^9^, four-qubit entangled states^10^, and three^8^ or two^12^ pairwise quantum key distribution (QKD) channels, and experimentally demonstrated using four photon-polarization entangled state^11^.

Interestingly, later works have shown that there are quantum solutions for certain communication complexity problems and secret sharing tasks which do not require entanglement, but, instead, sequential communication of a single quantum system^13^,^14^. These protocols have been shown to be much more resistant to noise and imperfections, and significantly more scalable than protocols based on entanglement.

In this article, we introduce a quantum algorithm that solves the DBA and achieves clock synchronization in the presence of an arbitrary number of faulty processes, with only one single round of message passing per process independently of the number of faulty processes, utilizing only a single quantum system.

In order to solve the DBA problem, the *m* processes need to share data in the form of lists *l_k_*, of numbers subject to specific correlations, and the distribution must be such that the list *l_k_* held by process *P_k_* is known only by *P_k_*. Quantum mechanics provides methods to generate and securely distribute such data, here we shall seek for one which is simple, efficient, and easily extendible to an arbitrary number of processes. We assume that all processes can communicate with one another with oral messages by pairwise authenticated error-free classical channels and pairwise authenticated quantum channels.

## Correlated lists and their use

The initial stage of the quantum protocol is to distribute lists *l_k_*, for *k* = 1, …, *m*, each of them available only to process *P_k_*. All lists have to be of the same length *L* and are required to satisfy the property that if *N* = 0 (or 1) is at position *j* in *l*_1_, then 0 (respectively, 1) is at position *j* in lists *l_k_* for *k* = 2, …, *m* (i.e., they are perfectly correlated). However, if *N* ∈ {2, …, *m* − 1} is at position *j* in *l*_1_, then the sum of numbers at positions *j* in lists *l_k_* for *k* = 2, …, *m* equals *m* − *N*, and all elements in these lists are either 0 or 1. Given an *N*, all the possible combinations of binary numbers satisfying the condition are uniformly probable.

Note that, on one hand, *P*_1_ has information about at which positions the lists of all other processes the values are perfectly correlated, and at which positions they are random bits, with the property that their sum is anticorrelated with the value, *N* ≥ 1, in *l_k_*. On the other hand, the holder of one the lists *l_k_*, with *k* = 2, …, *m*, has no information whatsoever on whether the lists are correlated at a given position or not.

Once the processes have these lists, they can use them to achieve mutual agreement and solve the DBA by applying the algorithmic part of the protocol, which we shall call *QB*(*n*, *m*). The special case, *QB*(1, 3), reproduces the protocol in^11^.

1. *P*_1_ sends bit-valued messages to all processes. The message sent to process *P_k_* will be denoted by *m*_1,*k*_. Together with each message, *P*_1_ sends a list *l*_1,*k*_ of all of the positions in *l*_1_ in which the value *m*_1,*k*_ appears. If *P*_1_ is nonfaulty all lists and messages are identical. The full information which *P_k_* receives from *P*_1_ will be denoted by {*m*_1,*k*_, *l*_1,*k*_}.
2. The receiving processes *P_k_* analyze (singlehandedly) the obtained lists and messages. If the analysis of *P_k_* shows that *l*_1,*k*_ is of appropriate length (i.e., about *L*/*m*) and {*m*_1,*k*_, *l*_1,*k*_} is consistent with *l_k_* at all positions, then if *P_k_* is nonfaulty, it conveys {*m*_1,*k*_, *l*_1,*k*_} to all other processes *P_k_*_≠1_. A faulty process sends a flipped bit value of the message with whatever list it chooses. The full information which *P_j_* receives from *P_k_* will be denoted by {*m_k_*_,*j*_, *l_k_*_,*j*_}.A nonfaulty *P_k_* will also decide on the final bit value it adopts *V_k_*. This is *m*_1,*k*_, unless messages from the other processes force it to decide that *P*_1_ is faulty. However, if {*m*_1,*k*_, *l*_1,*k*_} is not consistent with *l_k_*, then *P_k_* immediately ascertains that *P*_1_ is faulty and relays to other processes neither 0 nor 1 but ⊥, meaning “I have received inconsistent data.”
3. Once all messages have been exchanged between *P*_2_, …, *P_m_*, each process considers the obtained data and acts according to the instructions in Table 1. The overall aim is, if *P*_1_ is nonfaulty, to have the same value of *V_k_* for all nonfaulty processes, or all of them aborting.

## Quantum protocol for distributing lists l_k_

**Figure 1:**
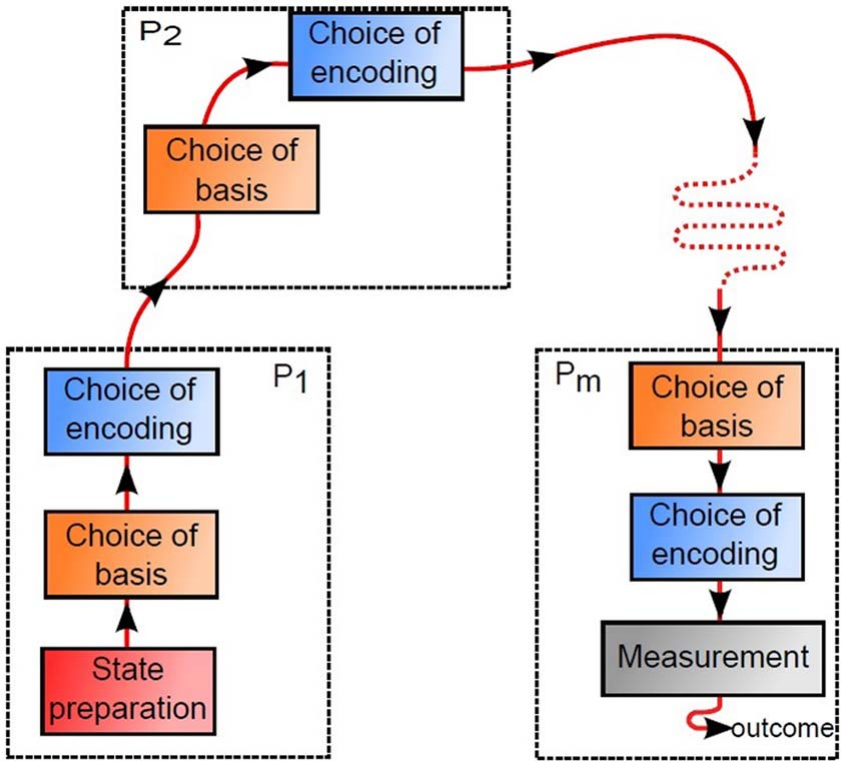
Scheme of the quantum protocol for the distribution of the correlated lists. *P*_1_ prepares a uniform *d*-level superposition state, makes a choice of basis and encoding, and forwards the qudit to *P*_2_ which applies a choice a basis and encoding and forwards the qudit to *P*_3_. Processes *P*_3_, …, *P_m_* act in analogy with *P*_2_. Finally *P_m_* projects the state onto the initial state prepared by *P*_1_ and, if the outcome is 1, the processes reveal their bases and, if all bases are the same, the round is treated as valid.

All processes are equipped with devices which can unitarily transform qudits. In addition, *P*_1_ has a source of *single qudits of dimension m* and the last process, *P_m_*, has *additionally* a measurement device. The protocol runs as follows (for an illustration, see Fig. 1):

- *P*_1_ prepares the state
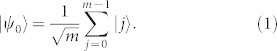
- *P*_1_ randomly chooses the “encoding basis” from *m* different options *U*_0_,...,*U*_*m*−1_ and labels the choice *c*_1_. Having chosen the *c*_1_’st encoding basis, process *P*_1_ applies the following unitary transformation to the qudit:
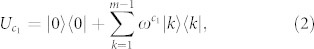
where 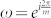. From the interferometric point of view, applying *U_c_1__* introduces a phase-shift of −2*πc*_1_/*m* in the first beam.
- After that, *P*_1_ randomly chooses a value *N*_1_ in the set {0, 1, …, *m* − 1} and encodes *N*_1_, by applying the following unitary transformation:
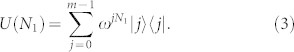
Afterwards, the qudit is sent to *P*_2_.
- *P*_2_, in the same manner as *P*_1_, choses a *c*_2_∈{0,...,*m*−1} and applies the unitary *U*_*c*_2__ corresponding to choice of encoding basis.
- Next, *P*_2_ randomly chooses a value *N*_2_ in the set {0, 1}. If *N*_2_ = 0, no action is taken, i.e., *P*_2_ applies the transformation 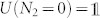. If *N*_2_ = 1, then *P*_2_ applies *U*(*N*_2_ = 1) and then sends the qudit to *P*_3_.
- *P*_3_, …, *P_m_* consecutively repeat the same procedure as *P*_2_ with independent choices of basis and encoding their respective random values *N*_3_, …, *N_m_*.
- In addition, *P_m_* measures the qudit using a device which distinguishes the state |*ψ*_0_〉 from any set of states orthogonal to it.
- If *P_m_* obtains |*ψ*_0_〉, then the processes consecutively reveal their encoding bases (but not their values *N_k_*) in reverse order: First *P_m_* and last *P*_1_. If it turns out that the sum of the basis choices modulo *m* equals zero, then the run is treated as a valid distribution of the numbers *N_k_* at the same position in the private lists *l_k_*.

The protocol distributes the numbers in the required way because all the unitary operators are diagonal and, therefore, commute. Additionally, if 
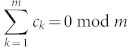
 then
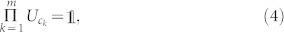
and, if 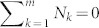, modulo *m*, then 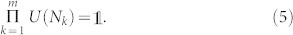


Whenever this condition is not satisfied, the final state of the system is orthogonal to |*ψ*_0_〉 and will therefore never be an outcome of *P_m_*'s measurement.

## Clock synchronization

Now, we will show how to apply our method for solving the DBA to achieve fault tolerant clock synchronization. However, a problem arises from clocks ticking during the synchronization procedure. This is solved by exploiting assumption A3: Instead of sending a number, the processes send their clock differences to each other. In the classical case, we achieve clock synchronization by running the algorithm *OM*(1) *m* times, sending clock differences instead of the binary values, and analogously for *OM*(*n*)^4^. In analogy with the classical case, the processes send clock differences also in the quantum case, exploiting the fact that the clock differences can be decomposed into binary strings up to arbitrary accuracy agreed upon in advance. We run *QB*(*n*, *m*) *m* times in such a way that for each run a new processes takes the roll of *P*_1_ in *QB*(*n*, *m*). More explicitly, *P_y_* reads the clock difference Δ*_xy_* between its own clock and the clock of *P_x_*. If *P_y_* is nonfaulty it will relay Δ*_xy_* to *P_z_* but if *P_y_* is a faulty process, it can arbitrarily change Δ*_xy_* before sending it. If *P_y_* relays the value obtained from *P_x_* to *P_z_*, then *P_z_* knows the time difference between *P_x_* and *P_y_*. Also, since *QB*(*n*, *m*) is ran *m* times, *P_z_* will also obtain Δ*_yz_* from *P_y_* and thus *P_z_* knows that *P_y_* is claiming that the time difference between *P_x_* and *P_z_* is Δ*_xy_* + Δ*_yz_*, which can then be compared to Δ*_xz_* obtained directly from *P_x_*.

## Comparison with the other solutions

The correlated lists needed for achieving DBA can be distributed by other means than with the single-qudit protocol. Successful distribution can be achieved by the process *P_m_* sharing a QKD channel with every other process. *P_m_* uses a QKD protocol, e.g., BB84^17^ to distribute numbers such that (1) *P_m_* and *P*_1_ share a string 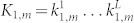, where 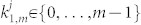. (2) For every *l* = 2, …, *m* − 1, *P_m_* and *P_l_* share a string 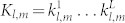 such that 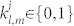. (3) For a given *j*, the lists satisfy 
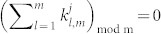
. (4) None of *P*_2_, …, *P_m_*_−1_ have any information about a particular list element of any other process. (5) Whenever *P*_1_ receives an element 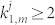, *P*_1_ has no information on the bit value of 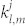 for *l* = 2, …, *m*, and whenever *P*_1_ receives 
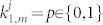
, *P*_1_ knows that 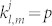 for all *l* = 2, …, *m*. All QKD channels except that shared between *P*_1_ and *P_m_* transmit bit values. In order to transmit elements of {0, …, *m* − 1} to *P*_1_, the numbers must be encoded into 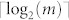 qubits. One additional requirement that has to be made for solving the DBA using the QKD distributed lists is that *P_m_* is not required to convey any lists. This is necessary since *P_m_* has full knowledge about the lists of all other processes and therefore easily could cheat. Instead, *P_m_* may announce the message it received from *P*_1_, and if any inconsistency is noted by *P*_2_, …, *P_m_*_−1_, then *P_m_* will change its final value if the other processes convince *P_m_* of them being nonfaulty.

There are also other proposed solutions to the DBA considering three processes where one is faulty. The first one, proposed in Ref. 7, relies on the three qutrit entangled Aharonov state. The goal is to distribute lists given by all permutations of the elements of the set {0, 1, 2}, i.e., (0–1–2, 0–2–1, 1–0–2, 1–2–0, 2–0–1, and 2–1–0). Generalization to *m* parties along the lines of Ref. 7 would require the usage of multipartite *m*-level entanglement, provided by the state 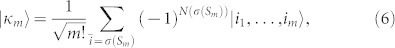 where 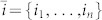, *S_m_* = {0, …, *m* − 1} and *N*(*σ*(*S_m_*)) is the parity of the permutation of *S_m_*. Already for the simplest case of *m* = 3, this approach requires the preparation of a very complex state which, to our knowledge, has not yet been experimentally realized. However, for the three process case, it has been pointed out in Ref. 12 that the distribution of the lists can be realized without the state (6), by utilizing two separated QKD channels. With small modification for the *m* process setting, distribution of the lists is achieved with *m* − 1 QKD channels. However, to encode the entire space provided by *S_m_*, the QKD requires 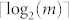 qubits. If the efficiency of a detector *η* is not perfect and the QKD is performed with single qubits using von Neuman measurements, successful distribution occurs only with probability 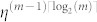. Typically, the classical part of the protocol in Ref. 7 and its possible generalizations scale rapidly with the number of processes. It is required that *m*! different types of lists are distributed. However, a solution to the three party DBA exploiting four-qubit entanglement provides a simpler classical part of the protocol: the number of different lists is lowered from six to four^11^.

The general *m* process protocol presented in this paper generalizes the protocol in Ref. 11 and requires 2*^m^*^−1^ different types of lists. As emphasized earlier, the distribution of the required lists can be achieved both with single-qudit and with *m* − **1** QKD channels. Using QKD channels, only one channel needs to transmit all elements in *S_m_* while the remaining *m* − 2 channels only transmit bit values. In the presence of nonperfect detectors, successful distribution occurs with probability 
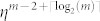
. However, in the single-qudit approach only one single detection is needed and, therefore, successful distribution of the lists occur with probability *η* independently of *m*. The single-qudit protocol is highly scalable, both in terms of success probability with inefficient detectors and requirements on the classical lists.

## Conclusions

We have presented a single-qudit protocol which provides an efficient solution to an important multiparty communication problem: It solves DBA and achieves clock synchronization in the presence of arbitrary many faulty clocks. In principle, our quantum algorithm is not limited to the case of clock synchronization, it can with small adaptation be used for other tasks requiring oral message interactive consistency. Interestingly, our algorithm works by transmitting a single qudit among the parties rather than by distributing a quantum entangled state among them. This makes the protocol much more practical, as single qudits can be experimentally realized easily in many ways. For example, using unbiased multiport beamsplitters^15^ or time-bin^16^. Compared to schemes based on several QKD channels, the single-qubit protocol is more scalable and robust against detection inefficiencies. This results shows that single-qudit quantum information protocols are interesting beyond QKD^18^,^19^ and random number generation^20^,^21^, and should stimulate experimental implementations and further research in quantum information protocols.

## Author Contributions

A.C., M.Z. and M.B. proposed and initiated the project. A.T. performed the analysis and the extension for n parties. All authors discussed the results, agreed on the conclusions, and wrote the manuscript.

## Figures and Tables

**Table 1:** Once *P_k_* receives all messages and lists from all other processes, it will study the obtained lists and messages and compare to its own list *l_k_*. Depending on the consistency between the obtained and private data, *P_k_* will act according to table below. Notation 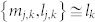 means that *m_j,k_* and *l_j,k_* are found to be consistent with *l_k_* whereas 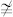 means “inconsistent with.” The symbol ⊥ means “I have received inconsistent data.” By 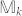 we denote some nonempty subset of {1, …, *m*} \ {*k*}

	local analysis of all data received by *P_k_*	decision of *P_k_* on the value *V_k_*
(iia)	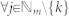 , 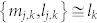 and all messages are equal	*V_k_* = *m*_1,*k*_, no faulty process
(iib)	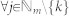 , 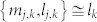 and *not* all messages are equal	as *P*_1_ is faulty, *V_k_* = *abort*
(iic)	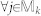 , 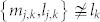 and 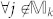 , 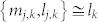	*V_k_* = *m_j,k_*, for 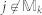 , as the other *P_j_*'s are faulty
(iid)	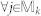 , 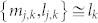 and 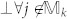	*V_k_* = *m*_1,*k*_, although *P*_1_ could be faulty
(iie)	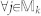 , 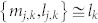 , but with unequal messages, and ⊥ from 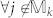	*V_k_* = *abort*, at least *P*_1_ is faulty
